# Changing positive and negative affects through music experiences: a study with university students

**DOI:** 10.1186/s40359-023-01110-9

**Published:** 2023-03-21

**Authors:** José Salvador Blasco-Magraner, Gloria Bernabé-Valero, Pablo Marín-Liébana, Ana María Botella-Nicolás

**Affiliations:** 1 Department of Music Education, University of Valencia, Av. Dels Tarongers, 4, 46022 Valencia, Spain; 2 Department of Occupational Sciences, Speech Therapy, Evolutionary and Educational Psychology, Catholic University of Valencia San Vicente Mártir, Av. De La Ilustración, 2, 46100 Burjassot, Valencia, Spain

**Keywords:** Emotion, Positive affects, Negative affects, Music experiences, University students

## Abstract

**Background:**

Currently, there are few empirical studies that demonstrate the effects of music on specific emotions, especially in the educational context. For this reason, this study was carried out to examine the impact of music to identify affective changes after exposure to three musical stimuli.

**Methods:**

The participants were 71 university students engaged in a music education course and none of them were musicians. Changes in the affective state of non-musical student teachers were studied after listening to three pieces of music. An inter-subject repeated measures ANOVA test was carried out using the Positive and Negative Affect Schedule (PANAS) to measure their affective state.

**Results:**

The results revealed that: (i) the three musical experiences were beneficial in increasing positive affects and reducing negative affects, with significant differences between the interaction of Music Experiences × Moment (pre-post); (ii) listening to Mahler’s sad fifth symphony reduced more negative affects than the other experimental conditions; (iii) performing the blues had the highest positive effects.

**Conclusions:**

These findings provide applied keys aspects for music education and research, as they show empirical evidence on how music can modify specific affects of personal experience.

**Supplementary Information:**

The online version contains supplementary material available at 10.1186/s40359-023-01110-9.

## Introduction

The studies published on the benefits of music have been on the increase in the last two decades [1–3] and have branched out into different areas of research such as psychology [4–8], education [1, 9, 10] and health [11, 12] providing ways of using music as a resource for people’s improvement.

The publication in 1996 of the famous report “Education Hides a Treasure” submitted to the UNESCO by the International Commission was an important landmark in the educational field. This report pointed out the four basic pillars of twenty-first century education: learning to know, learning to do, learning to live together, and learning to be [13]. The two last ones clearly refer to emotional education. This document posed a challenge to Education in terms of both academically and emotionally development at all levels from kindergarten to university. In this regard, there has been a notable increase in the number of studies that have shown the strong impact of music on the emotions in the different stages of education and our lives. For example, from childhood to adolescence, involving primary, secondary and university education, music is especially relevant for its beneficial effects on developing students’ emotional intelligence and prosocial skills [1, 14]. In adults, music benefits emotional self-regulation [15], while in old age it helps to maintain emotional welfare and to experience and express spirituality [16]. This underlines the importance of providing empirical evidence on the emotional influence of music.

### Influence of music on positive affects

Numerous studies have used the Positive and Negative Affect Schedule (PANAS) to evaluate the emotional impact of music [17]. This scale is valid and effective for measuring the influence of positive and negative effects of music on listeners and performers [10, 18, 19]. Thus, for example, empirical evidence shows that exposure to a musical stimulus favours the increase of positive affects [20, 21] found a significant increase in three positive affects in secondary school students after listening to music, and the same results has been found after listening to diverse musical styles. These results are consistent with Schubert [22], who demonstrated that music seems to improve or maintain well-being by means of positive valence emotions (e. g. happiness, joy and calm). Other research studied extreme metal fans aged between 18 and 34 years old and found statements of physiological excitement together with increased positive affects [21]. Positive outcomes after listening to sad music have also been found [23], who played Samuel Barbers’ *Adagio for Strings*, described by the BBC as the world’s saddest piece of classical music, to 20 advanced music students and 20 advanced psychology students with no musical background and subsequently found that the music only had positive affects on both groups.

Several experimental designs that used sad music on university students noticed that they experienced both sadness and positive affects [24, 25] and also found that music labeled as “happy” increased positive affects while the one labeled as “sad” reduced both positive and negative affects [26]. For other authors the strongest and most pleasant responses to sad music are associated with empathy [27]. Moreover, listening to sad music had benefits since attributes of empathy were intensified [27, 28]. In relation to musical performances, empirical evidence found a significant increase in positive affects [29]. Thus, music induces listeners to experience positive affects, which could turn music into an instrument for personal development.

Following on from Fredrickson’s ‘broaden‐and‐build’ framework of positive emotions [30], positive affects cause changes in cognitive activities which, in turn, can cause behaviour changes. They can also expand the possibilities for action and improve physical resources. According to Fredrickson [30], positive affects trigger three sequential effects: (1) amplification of the scope for thought and action; (2) construction of personal resources to deal with difficult simplifications; (3) personal transformation by making one more creative, with a better understanding of situations, better able to face up to difficulties and better socially integrated. This leads to an “upward spiral” in which even more positive affects are experienced. A resource such as music that can increase positive affects, can therefore be considered as a step forward in personal transformation. Thus, music teachers could have a powerful tool to help students enhance their personal development.

### Influence of music on negative affects

There is a great deal of controversy as regards the influence of music on negative affects. Blasco and Calatrava [20] found a significant reduction of five negative affects in secondary school students after listening to Arturo Marquez’s typically happy *Danzón N*^*O*^* 2.* Different results were found in an experiment in which the change in participants ‘affects was assessed after listening the happy "Eye of the Tiger" by Survivor and the sad "Everybody Hurts" by REM [26]. They found that the happy piece only increased the positive affects but did not reduce the negative ones, while the sad piece reduced both positive and negative affects. However, neither of these findings agree with Miller and Au [31], who carried out an experiment to compare the influence of sad and happy music on undergraduates ‘mood arousal and found that listening to both types had no significant changes on negative affects. Shulte [32] conducted a study with 30 university students to examine the impact that nostalgic music has on affects, and found that after listening to different songs, negative affects decreased. Matsumoto [33] found that sad music reduced sad feelings in deeply sad university students, while Vuoskoski and Eerola [34] showed that sad music could produce changes in memory and emotional judgements related to emotions and that experiencing music-induced sadness is intrinsically more pleasant than sad memories. It therefore seems that reducing negative affects has mostly been studied with sad and nostalgic musical stimuli. In this way, if music can reduce negative affects, it can also be involved in educational and psychological interventions focused on improving the emotional-affective sphere. Thus, for example, one study examined the effects of a wide range of music activities and found that it would be necessary to specify exactly what types of music activity lead to what types of outcomes [2]. Moore [3] also found that certain music experiences and characteristics had both desirable and undesirable effects on the neural activation patterns involved in emotion regulation. Furthermore, recent research on university students shows that music could be used to assess mood congruence effects, since these effects are reactions to the emotions evoked by music [35].

These studies demonstrate that emotional experience can be actively driven by music. Moreover, they synthesize the efforts to find ways in which music can enhance affective emotional experience by increasing positive affects and reducing the negative ones (e. g. hostility, nervousness and irritability). Although negative emotions have a great value for personal development and are necessary for psychological adjustment, coping with them and self-regulation capacities are issues that have concerned psychology. For example, Emotional Intelligence [36], which has currently been established in the educational field, constitutes a fundamental conceptual framework to increase well-being when facing negative emotions, providing keys for greater control and management of emotional reactions. It also establishes how to decrease the intensity and frequency of negative emotional states [37], providing techniques such as mindfulness meditation that have proven their effectiveness in reducing negative emotional experiences and increasing the positive ones [38]. The purpose of this research is to find whether music can be part of the varied set of resources that can be used by a teacher to modify students’ emotional experience.

Thus, although empirical evidence of the effects of music on the emotional sphere is still incipient. It seems that they can increase positive effects, but it is not clear their impact on the negative ones, since diverse and contradictory results (no change and reduction of negative affects after listening to music) were found. In addition, the effects of the type of musical piece (e.g. happy or sad music) need further investigation as different effects were found. Moreover, previous studies do not compare between the effects of listening to versus performing music. Such an approach could provide keys to highlight the importance of performing within music education. Therefore, this study aims to contribute to this scientific field, providing experimental evidence on the effects of listening to music as compared to performing music, as well as determining the effects of different types of music on positive and negative affects.

To this end, the effects of three different types of music experiences were compared: (1) listening to a sad piece, (2) listening to an epic and solemn piece, and (3) performing of a rhythm and a blues piece, to determine whether positive and negative affects were modified after exposure to these experimental situations. In particular, two hypotheses guided this study: (1) After exposure to each musical experience (listening to a sad piece; listening to a solemn piece and playing a blues), all participants will improve their emotional experience, increasing their positive affects and reducing their negative ones; and (2) the music performance will induce a greater change as compared to the listening conditions.

## Method

### Participants

A total of 71 students were involved in this study, 6 men and 65 women between the ages of 20 and 40, who were studying a Teaching Grade. These students were enrolled in the "Music Education" program as part of their university degree’s syllabus. None of them had special music studies from conservatories, academies or were self-taught; thus, all had similar musical knowledge. None of them had previously listened to music in an instructional context nor had performed music with their fellow students. In addition, none of them had listening before to the musical pieces selected for this experiment.

All signed an informed consent form before participating and no payment was given for taking part in the study. As the experiment was carried out in the context of a university course, they were assured that their participation and responses would be anonymous and would have no impact on their qualifications. The research was approved by the ethical committee at the Universidad Católica de Valencia San Vicente Mártir: UCV2017- 18-28 code.

### Materials

#### Questionnaire

To assess emotional states, the Positive and Negative Affective States scales (PANAS), was administered [39]. In particular, the Spanish version of the scale [17], whose study shows a high degree of internal consistency; in males 0.89 in positive affects and 0.91 in negative affects; in women 0.87 in positive affects and 0.89 in negative affects. In this study, good reliability level in each experimental condition was obtained (0.836–0.913 for positive affects and 0.805–0.917 for negative affects (see Table 1 for more information on Cronbach’s α for each experimental condition).

**Table 1:** Descriptive statistics and Cronbach's α

Condition	Mean	Std. deviation	Cronbach’s Alpha
Pre MML POS	31.5519	6.19292	0.836
Post MML POS	30.0069	6.11794	0.853
Pre VML POS	27.8732	5.98075	0.883
Post VML POS	27.1812	6.11978	0.894
Pre BP POS	29.2034	6.28141	0.894
Post BP POS	22.3056	6.45341	0.913
Pre MML NEG	41.7326	7.22804	0.843
Post MML NEG	45.9982	6.14585	0.917
Pre VML NEG	42.7042	5.72811	0.879
Post VML NEG	45.3261	5.00389	0.884
Pre BP NEG	44.6897	4.65231	0.823
Post BP NEG	45.9993	3.91162	0.805

Score obtained in the PANAS in each experimental condition

The PANAS consists of 20 items which describe different dimensions of emotional experience. Participants must answer them regarding to their current affective state. The scale is composed of 20 items; 10 positive affects (PA) and 10 negative affects (NA). Answers are graded in a 5-options (Likert scale), with reversed items, ranging from extremely (1) to very slightly or not at all (5).

#### Musical pieces

The musical pieces choice stemmed from the analysis of some of the music elements that most influence the perception of emotions: mode, melody and intervals. Within the melody, range and melodic direction were distinguished. The range or amplitude of the melodic line is commonly divided into wide or narrow, while the melodic direction is often classified as ascending or descending. Chang and Hoffman [10] associated narrow amplitude melodies with sadness, while Schimmark and Grob [40] related melodic amplitude with highly activated emotions. Regarding the melodic direction, Gerardi and Gerken [41] found a relationship between ascending direction and happiness and heroism, and between descending direction and sadness.

In relation to the mode, Tizón [42] stated that the major one is completely happy, while the minor one represents sadness. Thompson and Robitaille [43] considered that, in order to cause emotions such as happiness, solemnity or joy, composers use tonal melodies, while to obtain negative emotions, they use atonality and chromaticism.

In this research, the selected pieces (“Adagietto” from Gustav Mahler's Fifth Symphony, MML; and “Titans” from Alexander The Great from Vangelis, VML) are representative examples of the melodic, intervallic and modal characteristics previously exposed. Mahler's and Vangelis's pieces completely differ in modes and melodic amplitude (sad vs. heroism). Likewise, Mahler's piece is much more chromatic than Vangelis' one, which has a broader melody made up of third, fourth and fifth intervals, often representative of heroism. Those features justify the fact that they have been used as soundtracks in two films belonging to the epic genre (Alexander The Great, 2004) and drama (Death in Venice, 1971).

The musical piece that was performed by the students was chosen in order to be easy to learn in a few sessions, since they were not musicians. So, three musical pieces were used for the experimental conditions, the first two musical pieces were recordings in a CD, while the third one was performed by the subjects.

The three chosen pieces are described below:

Condition 1 (MML): “Adagietto” from Gustav Mahler’s Fifth Symphony (9:01 min), performed by the Berlin Philharmonic conducted by Claudio Abbado [44]. This is a sad, melancholic and dramatic piece that Luchino Visconti used in the film Death in Venice, made in 1971 and based on the book by Thomas Mann.

Condition 2 (VML): “Titans Theme” from Alexander the Great (3:59 min), directed by Oliver Stone and premiered in 2004, whose music was composed, produced and performed by Vangelis [45]. It has a markedly epic character with large doses of heroism and solemnity.

Condition 3 (BP): “Rhythm’s Blues” composed and played by Ana Bort (4:00 min). This is a popular African-American piece of music with an insistent rhythm and harmonically sustained by tonal degrees. This piece was performed by the participants using percussion instruments (carillons and a range of xylophones and metallophones).

### Procedure

The sample was divided into two groups (N_1_ = 36 and N_2_ = 35) that participated separately in all the phases of the study. The first two conditions (MML and VML) were carried out in each group's classroom, while the performance (BP) was developed in the musical instruments room. This room had 52 percussion instruments, including different types of chimes, xylophones and metallophones (soprano, alto and bass). It is a large space where there are only chairs and musical instruments and stands. The first group was distributed as follows: 6 chimes (3 soprano and 3 alto), 5 soprano xylophones, 5 alto xylophones, 5 bass xylophones, 5 soprano metallophones, 5 alto metallophones and 5 bass metallophones. The distribution of the second group was similar, but with one less alto metallophone.

Prior to the experiment, participants received two practical lessons in order to learn how to collectively perform the music score (third experimental condition). After the two practical lessons, during the next three sessions (leaving two weeks between each session), the experiment was carried out. In each session, an experimental condition was applied and PANAS was on-line administered online beforehand and afterwards (Pre-Post design). All participants were exposed to the three experimental conditions and completed the scale before and after listening to music.

In each of these three sessions, a different music condition was applied: MML in the first one, VML in the second one and BP in the third one.

As conditions VML and MML were listening to pieces of music, the instructions received by the subjects were: “You are going to listen to a musical piece, you ought to listen actively, avoiding distractions. You can close your eyes if you feel like to”. For the BP condition, they were said to play the musical sheet all together.

The aim of the study was to examine the effect of the music experience variable (with three levels: MML, VML and BP) in the Positive and Negative Affects subscales from the PANAS scale. The variable Moment was also studied to control biases and to analyze differences between the Pre and Post conditions.

### Design

The experiment was designed as a two-way repeated measure (RM) ANOVA with two dependent variables: Positive Affects and Negative Affects, one for each PANAS’ subscales.

The two repeated measures used in the experiment were the variables Musical Experience (ME), with three levels (MML, VML and BP) and the variable Moment, with two levels (PRE and POST). All participants were exposed to the three experimental conditions.

The design did not include a control group, similar to many other studies in the field of music psychology [27, 30]. The control was carried out from the intra-subject pre-post measurement of all the participants. The rationale for this design lies in the complexity of the control condition (or placebo) design in psychology [46]. While placebos in pharmacological trials are sugar pills, in psychology it is difficult to establish an equivalent period of time similar to the musical pieces (e. g. 9 min) without activity, so that cognitive activity occurred during this period of time (e. g. daydreaming, reading a story, etc.) could bias and limit the generalization of results.

Additionally, one of the goals of this study was to compare the effects of listening to music compared to performance on affects. For this reason, two music listening experiences (MML and VML) and a musical performance experience (BP) were designed. In order to control potential biases, participants did not know the musical pieces in the experimental conditions and they had a low level of musical performance competence (musicians were excluded).

### Analysis

It was used SPSS statistics v.26 for the statistical analyzes.

Two ANOVA were performed. The first one, analyzed two dependent variables at the same time: Positive Affects (PA) and Negative Affects (NA).

In the second ANOVA, the 20 items of the PANAS scale were taken as dependent variables. The rest of the experimental design was similar to the first one, a two-way RM ANOVA with variables Musical Experience (ME) and Moment as repeated measures.

## Results

Examination of frequency distributions, histograms, and tests of homogeneity of variance and normality for the criterion measures indicated that the assumptions for the use of parametric statistics were met. Normality was met in all tests except for one, but the ANOVA is robust against this assumption violation. All the analyses presented were performed with the significance level (alpha) set at 0.05, two-tailed tests. Means and standard deviations for the 6 experimental conditions for both subscales, Positive Affects and Negative Affects, are presented in Table 1.

Mauchly’s test of sphericity was statistically significant for Musical Experience and Musical Experience*Moment focusing on NA as the dependent variable (*p* < 0.05). The test only was significant for Musical Experience for PA as dependent variable (*p* < 0.05). The rest of the W’s Mauchly were not significant (*p* > 0.05), so we assumed sphericity for the non-mentioned variables and worked with the assumed sphericity univariate solution. For the variables which the W’s Mauchly was significant, the univariate solution was also taken, but choosing the corrected Greenhouse–Geisser epsilon approximation due to its conservativeness.

A significant principal effect of the Musical Experience variable F(1.710,119.691) = 22.505, *p* < 0.05, η^2^ = 0.243; the Moment variable F(1,70) = 45.291, *p* < 0.05, η^2^ = 0.393; and the Musical Experience*Moment interaction F(2,140) = 32.502, *p* < 0.05, η^2^ = 0.317 were found for PA.

Statistically significance was found for Moment F(1, 70) = 70.729, *p* < 0.05, η^2^ = 0.503 and Musical Experience*Moment interaction F(1.822, 127.555) = 8.594, *p* < 0.05, η^2^ = 0.109, but not for Musical Experience F(1.593, 111.540) = 2.713, *p* < 0.05, η^2^ = 0.037, for the other dependent variable, NA.

Table 2 shows pairwise comparisons between Musical Experience levels. Bonferroni’s correction was applied in order to control type I error. We only interpret the results for the Positive Affects because the Musical Experience effect was not statistically significant for Negative Affects. Results show that condition VML presents a significant higher punctuation in Positive Affects than the other two conditions (*p* < 0.05). It also shows that the musical condition MML is significantly above BP in Positive Affects (*p* < 0.05).

**Table 2:** Pairwise comparisons for music experience

DV	(I) Music experience	(J) Music experience	Mean difference (I–J)	Std. error	p	95% Confidence interval for difference^a^	
						Lower bound	Per bound
PA	1	2	− 3.252*	.853	.001	− 5.345	− 1.159
		3	1.773*	.585	.010	.337	3.208
	2	1	3.252*	.853	.001	1.159	5.345
		3	5.025*	.813	.000	3.031	7.019
	3	1	− 1.773*	.585	.010	− 3.208	− .337
		2	− 5.025*	.813	.000	− 7.019	− 3.031
NA	1	2	.150	.740	1.000	− 1.665	1.964
		3	− 1.329*	.502	.030	− 2.560	− .099
	2	1	− .150	.740	1.000	− 1.964	1.665
		3	− 1.479	.817	.223	− 3.482	.524
	3	1	1.329*	.502	.030	.099	2.560
		2	1.479	.817	.223	− .524	3.482

Based on estimated marginal means

*The mean difference is significant at the 0.05 level

^a^Adjustment for multiple comparisons: Bonferroni

As regards Moment variable (Table 3), all but one Pre-Post differences were statistically significant (*p* < 0.05) for all the three conditions for both Positive and Negative Affects dependent variables. The Pre-Post difference found in Positive Affects for the VML Musical Experience did not reach the statistical level (*p* = 0.319).

**Table 3:** Pairwise Comparisons for PRE-POST in each music experience

DV	Music experience	(I) Moment	(J) Moment	Mean Difference (I–J)	Std. Error	p	95% confidence Interval for Difference^a^	
							Lower bound	Upper bound
PA	1	1	2	.692	.689	.319	− .683	2.067
		2	1	− .692	.689	.319	− 2.067	.683
	2	1	2	1.545*	.628	.016	.293	2.797
		2	1	− 1.545*	.628	.016	− 2.797	− .293
	3	1	2	6.898*	.664	.000	5.573	8.223
		2	1	− 6.898*	.664	.000	− 8.223	− 5.573
NA	1	1	2	− 2.622*	.585	.000	− 3.788	− 1.456
		2	1	2.622*	.585	.000	1.456	3.788
	2	1	2	− 4.266*	.558	.000	− 5.379	− 3.152
		2	1	4.266*	.558	.000	3.152	5.379
	3	1	2	− 1.310*	.416	.002	− 2.140	− .479
		2	1	1.310*	.416	.002	.479	2.140

Based on estimated marginal means

*The mean difference is significant at the 0.05 level

^a^Adjustment for multiple comparisons: Bonferroni

Focusing on these statistically significant differences, we observe that conditions MML and BP, for PA, decreased from Pre to Post condition, indicating that positive emotions increased significantly between pre and post measures. On the other hand, for NA, all conditions increased from Pre to Post conditions, indicating that negative affects were decreased between pre and post conditions. Once again, one should bear in mind that items were reversed, thus, a higher scores in NA means a decrease in affects.

In order to measure the interaction effect, significant differences between simple effects were analysed.

The simple effect of Moment (level2-level1) in the first Music Experience condition (MML) in PA was compared with the simple effect of Moment (level2-level1) in the second Musical Experience condition (VML). Music Experience conditions 2–3 (VML-BP) and 1–3 (MML-BP) were compared in the same way. Thus, taking into account PA and NA variables, a total of 6 comparisons, 3 per dependent variable, were made.

The results of these comparisons are shown in Table 4. Comparisons for PA range from T1 to T3 and comparisons for NA range from T4 to T6. All of them are significant (*p* < 0.05) which means that there are statistically significant differences between all the Musical Experience conditions when comparing the Moment (pre/post) simple effects.

**Table 4:** ANOVA of the interaction music experience × moment

	Contrast	Sum of squares	df	Mean square	F	*p*	Partial Eta squared
Interaction	T1	355.280	1	355.280	5.122	.027	.068
	T2	4,089.970	1	4089.970	49.211	.000	.413
	T3	5,060.890	1	5060.890	70.908	.000	.503
	T4	3,368.053	1	3368.053	74.112	.000	.514
	T5	1,097.395	1	1097.395	26.930	.000	.278
	T6	2,206.912	1	2206.912	55.197	.000	.441
Error	T1	4,855.618	70	69.366			
	T2	5,817.784	70	83.111			
	T3	4,996.077	70	71.373			
	T4	3,181.200	70	45.446			
	T5	2,852.464	70	40.749			
	T6	2,798.796	70	39.983			

T1: Comparison between the simple effect of Moment (level2-level1) in the first Music Experience condition (MML) with the simple effect of Moment (level2–level1) in the second Music Experience condition (VML) in PA. T2: the similar between VML and BP in PA. T3: the similar between MML and BP in PA. T4: the similar between MML and VML in NA. T5: the similar between VML and BP in NA. T6: the similar between MML and BP in NA

In Table 5, we can look at the differences’ values. As we said before the differences between Pre and Post conditions are significant when comparing the three musical conditions. The biggest difference for positive affects is between MML and BP (T3 = 8.443), and between VML and MML (T4 = − 6.887) for negative affects.

**Table 5:** Pairwise comparisons for music experience *moment

	Contrast					
	T1	T2	T3	T4	T5	T6
Contrast estimate	2.237	7.590	8.443	− 6.887	− 3.931	− 5.575
Hypothesized Value	0	0	0	0	0	0
Difference (Estimate—hypothesized)	2.237	7.590	8.443	− 6.887	− 3.931	− 5.575
Std. error	.988	1.082	1.003	.800	.758	.750
*P*	.027	.000	.000	.000	.000	.000
*95% confidence interval for difference*						
Lower bound	.266	5.432	6.443	− 8.483	− 5.442	− 7.072
Upper bound	4.208	9.748	10.442	− 5.292	− 2.420	− 4.079

Effects of the interaction between music experience × moment

In this second part, the results obtained from the second two-way RM ANOVA with the 20 items as dependent variables are considered. Results of the descriptive analysis of each item: *Interested, Excited, Strong, Enthusiastic, Proud, Alert, Inspired, Determined, Attentive, Active, Distressed, Upset, Guilty, Afraid, Hostile, Irritable, Ashamed, Nervous, Jittery, Scared*; in each musical condition: MML, VML and BP; and for the PRE and POST measurements, can be found in the Additional file 1 (Appendix A).

As regards the ANOVA test that compares the three experimental conditions in each mood, Mauchly’s Sphericity Test indicates that sphericity cannot be assumed for the musical experience in most of the variables of the items of effects, except for *Interested, Alert, Inspired, Active* and *Irritable*. For these items, the highest observed power index among Greenhouse–Geisser, Huynh–Feldt and Lower-bound epsilon corrections was taken for each variable. For the interaction Musical Experience*Moment, sphericity was not assumed for *Distressed, Guilty, Hostile* and *Scared*. For these items, the same above-cited criterion was followed.

Musical experience has a principal effect on all the positive affects, but only has it for 5 negative affects (*Nervous, Jittery, Scared, Hostile* and *Upset*) (*p* < 0.05). For more detail see Table S1 from Additional file 1: Appendix B.

The principal effect of Moment is also statistically significant (*p* < 0.05) for all (positive and negative), but two items: *Guilty* (*p* = 0.073) and *Hostile* (*p* = 0.123). All the differences between Pre and Post for positive affects are positive, which means that scores in conditions Pre were significantly higher than in condition Post. The other way around occurs for negative affects, all the differences Pre-Post are negative, meaning that the Post condition is significantly higher than the Pre condition. For more detail, see Table S2 from Additional file 1: Appendix B. In this way, Pre-post changes (Moment) improve affective states; the positive affects increase while the negative are reduced, except for *Guilty* (*p* = 0.073) and *Hostile* (*p* = 0.123).

Comparing the proportion of variance explained by the musical experienced and Moment (Tables s1 and s2 from the Additional file 1: Appendix B), it is observed that most of the η^2^ scores in musical experience are below 0.170, except *Active* and *Alert*, which are higher. On the other hand, the η^2^ scores for Moment are close to 0.300. From these results we can state that, taking only one of the variables at a time, the proportion of the dependent variable’s variance explained by Moment is higher than the proportion of the dependent variable’s variance explained by Musical Experience.

The effect of interaction, shown in Table S3 from the Additional file 1: Appendix B is significant in 7 positive moods (*Interested, Excited, Enthusiastic, Alert, Determined, Active* and *Proud*) and 4 negative moods (*Hostile*, *Irritable, Nervous*, and *Jittery*).

The pairwise comparisons of Musical Experience’s levels show a wide variety of patterns. Looking at Positive Affects, there is only one item (*Active*) which present significant differences between the three musical conditions. Items *Concentrated* and *Decided* do not present any significant difference between any musical conditions. The rest of the Positive items show at least one significant difference between conditions VML and BP. All differences are positive when comparing VML-MML, VML-BP MML-BP, except for Alert and Proud. So, in general, scores are higher for the first two conditions in relation to the third one, meaning that third musical condition presents the biggest increase for Positive Affects (remember items where reversed). For more detail see Additional file 1: Appendix C.

As regard pairwise comparisons of Musical Experience’s for negative affects, only the items which had a significant principal effect of the variable Musical Experience are shown here. There is a significant difference between conditions VML and MML in item *Nervous*; between VML and BP for *Scared* (*p* < 0.05). For *Jittery*; all three conditions differed significantly from each other (*p* < 0.05). Conditions MML and BP differed significantly for *Hostile* (*p* < 0.05) and conditions VML and BP almost differed significantly for *Upset* item, but null hypothesis cannot be rejected as *p* = 0.056. For more detail see Additional file 1: Appendix C. All differences were negative when comparing VML-MML, VML-BP MML-BP, except for *Nervous* and *Jittery*. So, in general, scores are lower for the first and second condition in relation to the third one.

## Discussion

Positive effects increased significantly during the post phase of all the music experiences, showing that exposure to any of the three music stimuli improved positive affectivity. There were also significant differences between the three experiences in this phase, according to the following order of improvements in positive affectivity: (1) the rhythm and blues performance (BP), (2) listening to Mahler (MML) and (3) listening to Vangelis (VML). As regards the effects of the *musical experience x Moment interaction*, all the comparisons were significant, with bigger differences in the interpretation of the blues (BP) than in listening to Mahler (MML) and Vangelis (VML). However, the comparison between both experiences, although significant, was smaller. These results indicate that performing music is significantly effective in increasing positive effects. We will explain these results in greater detail below as regards the specific affective states.

As regards Negative Affects, the comparison of the simple effects showed that these decreased after the musical experiences, although in this first analysis the VML musical experience did not differ from the other two. However, the results of the effects of the interaction between *musical experiencie x Moment* showed that all the comparisons were significant, with a larger difference between MML and VML than the one between BP and each of the other experiences. Listening to Mahler (MML) was more effective in reducing negative affects, compared to both listening to Vangelis and interpreting the blues (BP). These results agree with previous studies [26, 32], in which listening to sad music helped to reduce negative affectivity. In this study, it was the most effective condition, although exposure to all three musical experiences reduced negative affects.

The analysis of the specific affective states shows that most items that belong to Positive Affect scale are the most sensitive ones to the PRE-POST change, the different musical conditions and the interpretation of both effects. However, some items of the Negative Affect scale did not differ in the different music conditions or in the music experience × Moment interaction*.* For example, there were two items *(Guilty* and *Hostile)* that did not obtain significance. These results are consistent with the fact that music has certain limits as regards its impact on people’s affects and does not influence all equally. For example, *Guilty* has profound psychological implications that cannot be affected by simple exposure to certain musical experiences. This means we should be cautious in inferring that music alone can have therapeutical effects on complex emotional states whose treatment should include empirically validated methods. Also, emotional experiences are widely diverse so that any instrument used to measure them is limited as regards the affective/emotional state under study. These results suggest the importance of reviewing the items that compose the PANAS scale in musical studies to adapt it in order to include affective states more sensitive to musical experiences and eliminate the least relevant items.

The analysis of the results in the specific affective states, allows us to delve deeper into each experimental condition. Thus, regarding the results obtained in the complete scale of PANAS, listening to Mahler (MML), causes desirable changes by raising two positive affects (*Inspired* and *Attentive*) and reducing 10 negative affects (*Distressed, Upset, Afraid, Hostile, Irritable, Ashamed, Nervous, Jittery,* and *Scared*). This shows that this music condition had a greater effect on the negative affects than the other ones. These results agree with previous studies [26, 32], which found that sad music could effectively reduce negative affects, although other studies came to the opposite conclusion. For instance, Miller and Au [31] found that sad music did not significantly change negative affects. Some authors [47, 48] have argued that adults prefer to listen to sad music to regulate their feelings after a negative psychological experience in order to feel better. Taruffi and Koelsch [49] concluded that sad music could induce listeners to a wide range of positive effects, after a study with 772 participants. In order to contribute to this debate. It would be interesting to control personality variables that might explain these differences on the specific emotions evoked by sad music. In this study, it has been shown that a sad piece of music can be more effective in reducing negative affects than in increasing positive ones. Although the results come from undergraduate students, similar outcomes could be obtained from children and adolescents, although further research is required. In fact, Borella et al. [50] studied the influence of age on the effects of music and found that the emotional effects influenced cognitive performance (working memory) in such a way that the type of music (Mozart vs. Albinoni) had a stronger influence on young people than on adults. Kawakami and Hatahira [28], in a study on 84 primary schoolchildren, also found that exposure to sad music pleased them and their level of empathy correlated with their taste for sad music.

Listening to Vangelis (VML) increased 3 positive affects (*Excited, Inspired* and* Attentive*) and reduced 8 negative affects (*Distressed, Upset, Afraid, Irritable, Ashamed, Nervous, Jittery*, and *Scared*). Surprisingly, two positive affects were reduced in this experimental condition (*Alert* and *Attentive*). It could be explained due to the characteristic ostinato rhythm of this piece of music. It was found a similar effect in the study by Campbell et al., [26] in which sad music reduced both positive and negative affects. This musical condition also managed to modify negative affects more than positive ones.

Performing the blues (BP) increased all 10 positive affects, indicating that performing is more effective in increasing positive affects than listening. These results agree with the study by Dunbar et al. [29], who found that music performance significantly increased positive affects.

Performing the blues (BP) reduced 6 negative affects, although it was more effective in increasing positive affective states. Vigorous rhythmic music was also found to be positively associated with the use of all the forms of regulating emotions, which suggests that this type of music is especially useful for emotion modulation [51]. It was found an exception, since *Jittery* increased after the blues performance. It could be explained by the negative experience that is sometimes associated with music performance. Therefore, it should be taken into account that music performance could increase some negative effects. For example, Dimsdale et al. [52] found that a strong negative emotional response to a certain type of music in adolescents was related to risk behaviour, indicating that research into the repertory of music experiences needs to be broadened to diverse styles in different age groups to identify all the types of emotional response and their psychological consequences. However, this result should be taken with caution and further research should focus on whether the effect of increased agitation is usual after music performances.

To sum up, this study contributes to the scientific field on the following points: (1) all the musical experiences had significant effects on improving emotional states, increasing positive affects and decreasing the negative ones, which shows the importance of musical experiences on improving the affective sphere; (2) the specific affects that increased, decreased or did not change for each musical experience were identified, providing specific and useful keys for the design of future interventions; and (3) the differences between various types of musical experiences were analyzed, finding more improvements in the performing conditions than in the listening ones.

## Limitations and future directions

### Limitations

The sample, made up of university students with a very homogeneous profile in terms of age and sociodemographic characteristics, could limit the generalization of the results. In addition, the low percentage of men in the sample could also affect the generalizability of the results, although no previous studies have reported gender-based differential effects on the positive and negative affects after musical experiences.

Besides, the choice of the pieces of music was based on theoretical criteria and students’ music preferences were not taken into account. This will be included in future research, since the specific choice of the pieces could affect the positive or negative valence of participants’ emotions. However, the goal of using pieces of music not chosen by participants was to elicit new musical experiences for them. Furthermore, no participant was a musician and none of them had previous knowledge of any of the pieces, which may lead to a bias in the results.

In relation to this, the huge amount of available pieces of music, all of them influenced by their cultural and historical context, make it difficult to generalize that certain music parameters correlate with specific emotions. It would be necessary a cross-cultural approach to reach that conclusion.

### Future directions

It is recommended to introduce the variables of music preferences and music history to control their effect on the results and to be able to compare the different musical parameters of the pieces together with participants’ preferences.

Likewise, it would be interesting to identify the affects with a greater or lesser degree of influence by music, to adjust the psychological evaluation instrument to the characteristics of the experiment, including items of emotions that can be modified after exposure to a music experience.

The PANAS manual [39] indicates that a wide variety of affective states (60) and eight different temporal instructions were included in its construction, showing its great versatility. In further research, this instrument should be adapted to for a more specific application to music studies. For instance, by including other emotional states that could be related with the influence of music (e.g. *Tranquility*, *Gratitude*, *Elevation*), in order to measure more exactly the effects of music on people’s affective experiences.

Accordingly, it would be interesting to evaluate participants' affective traits to establish a baseline and control personality variables, helping to delve into the different levels of the hierarchical structure of affectivity and its relationship with the various music parameters.

Finally, it is recommended that the psychology of music include objective psychophysiological measurements together with self-report evaluations, so that conclusions arising from the experiments have greater robustness and can increase the impact of the contribution to the scientific community.

## Conclusion

This study have shown how different music experiences, such as listening and performing, influence the changes in positive and negative affects in student teachers. The results show that the three musical experiences studied are effective in improving the affects by comparing the emotional states before and after the music experiences. It was also showed that there are differences between the effects obtained in each of the music experiences. Besides, improving both types of affects will depend largely on the selected music for the purpose. Although further evidence is required, the results support the importance of music in education, since it provides tools to increase positive affects and to decrease the negative ones, which is important for emotional intelligence development [53, 54].

The three music experiences studied are more effective in reducing negative emotional states than in increasing the positive ones. This finding provides useful clues for music teachers to provide strategies that favor emotional regulation. For instance, in order to reduce hostility, irritability and nervousness, students could be exposed to musical auditions of both sad and solemn pieces, choosing musical pieces with similar characteristics to those described in this study. These auditions will be a resource for stress management in the classroom, as well as a tool that students can adopt and generalize to other contexts. Moreover, it is highly likely that students have not heard this type of music before and this experience could increase their repertoire of musical preferences, enhancing their emotional regulation.

The blues performance had a greater impact on participants' positive affects than listening to the other two pieces so, if any teacher wants to increase them (e.g., enthusiasm, interest, etc.), students could be asked to perform simple pieces such as Rhythm's Blues. In this way, musical performance could increase students' resources, contributing to higher levels of motivation, concentration and interest, which promotes learning [55–58]. Likewise, it could be very useful for elementary and secondary music teachers, who will be able to contribute to socio-emotional improvement and personal development of their students. Particularly, musical experiences could be a valuable resource for secondary teachers, since music is important in adolescents' lives and can be an interesting tool for meeting their emotional needs [59]. This is supported by Kokotsaki and Hallam [60], who consider that performing music helps students feel like active agents of a group, develop a strong sense of belonging, gain popularity, make "like-minded" relationships, improve their social skills and foster a strong sense of self-esteem and satisfaction.

This study shows that experiencing with various unknown musical pieces can have positive effects on emotions. According to this finding, university professors of Teaching grade in music education should encourage future teachers to experience various musical styles, rhythms and tonalities, avoiding prejudices. Thereby, future music teachers will be able to use a diversity of musical experiences that broaden the emotional effects and fulfill the socio-emotional function of music education. In relation to Fredrickson's 'broaden‐and‐build' framework of positive emotions [30], music can become a mean of widening other positive emotional states, constructing personal resources and transforming people, and contribute to an upward spiral of positive emotions. Taking into account the underlying psychological mechanisms of the impact of music on the emotional states it will be possible to use it to improve emotional area and other aspects of the personal sphere, as Chang et al., [10] maintain. Therefore, music education is an important resource to improve the emotional development of students.

## Supplementary Information

**Additional file 1**. Results obtained from item analyses.

## Data Availability

The datasets generated during and/or analyzed during the current study are available from the corresponding author on reasonable request.
