# Supplementary material for: Changing positive and negative affects through music experiences: a study with university students
Source: BMC Psychol. 2023 Mar 21;11:76. doi: 10.1186/s40359-023-01110-9 (PMC10031901; doi:10.1186/s40359-023-01110-9)
Supplement: Supplementary file 1 — Additional file 1. Results obtained from item analyses. [file 40359_2023_1110_MOESM1_ESM.docx]

Supplementary Material

Appendix A

| **Table 1**  *Descriptive Statistics* | | | |
| --- | --- | --- | --- |
|  | Mean | Standard Deviation |  |
| PRE MML Interested | 3.01 | .902 |  |
| POST MML Interested | 2.89 | .788 |  |
| PRE VML Interested | 2.89 | .832 |  |
| POST VML Interested | 2.92 | .781 |  |
| PRE BP Interested | 2.84 | .812 |  |
| POST BP Interested | 2.13 | .818 |  |
| PRE MML Excited | 3.16 | 1.008 |  |
| POST MML Excited | 3.05 | .894 |  |
| PRE VML Excited | 2.99 | .831 |  |
| POST VML Excited | 2.73 | .804 |  |
| PRE BP Excited | 3.00 | .864 |  |
| POST BP Excited | 2.15 | .858 |  |
| PRE MML Strong | 3.13 | .984 |  |
| POST MML Strong | 3.02 | 1.029 |  |
| PRE VML Strong | 2.88 | .866 |  |
| POST VML Strong | 2.75 | .924 |  |
| PRE BP Strong | 2.82 | .937 |  |
| POST BP Strong | 2.49 | .883 |  |
| PRE MML Enthusiastic | 3.36 | 1.037 |  |
| POST MML Enthusiastic | 3.15 | .936 |  |
| PRE VML Enthusiastic | 2.89 | .766 |  |
| POST VML Enthusiastic | 2.75 | .780 |  |
| PRE BP Enthusiastic | 3.00 | .813 |  |
| POST BP Enthusiastic | 2.27 | .884 |  |
| PRE MML Proud | 2.56 | .912 |  |
| POST MML Proud | 2.44 | .981 |  |
| PRE VML Proud | 2.55 | .905 |  |
| POST VML Proud | 2.48 | .870 |  |
| PRE BP Proud | 2.46 | .687 |  |
| POST BP Proud | 2.03 | .696 |  |
| PRE MML Alert | 3.26 | 1.032 |  |
| POST MML Alert | 3.38 | 1.077 |  |
| PRE VML Alert | 2.07 | .825 |  |
| POST VML Alert | 2.39 | .833 |  |
| PRE BP Alert | 2.96 | .958 |  |
| POST BP Alert | 1.77 | .803 |  |
| PRE MML Inspired | 3.62 | 1.039 |  |
| POST MML Inspired | 3.05 | .940 |  |
| PRE VML Inspired | 3.19 | .878 |  |
| POST VML Inspired | 2.90 | .770 |  |
| PRE BP Inspired | 3.17 | .896 |  |
| POST BP Inspired | 2.50 | 1.005 |  |
| PRE MML Determined | 2.94 | .954 |  |
| POST MML Determined | 2.98 | .880 |  |
| PRE VML Determined | 2.80 | .781 |  |
| POST VML Determined | 2.72 | .968 |  |
| PRE BP Determined | 2.98 | .895 |  |
| POST BP Determined | 2.46 | .852 |  |
| PRE MML Attentive | 3.24 | .911 |  |
| POST MML Attentive | 2.83 | .866 |  |
| PRE VML Attentive | 3.07 | .884 |  |
| POST VML Attentive | 2.66 | .854 |  |
| PRE BP Attentive | 3.05 | .801 |  |
| POST BP Attentive | 2.47 | .884 |  |
| PRE MML Active | 3.27 | 1.008 |  |
| POST MML Active | 3.21 | .870 |  |
| PRE VML Active | 2.55 | .936 |  |
| POST VML Active | 2.88 | .820 |  |
| PRE BP Active | 2.93 | 1.020 |  |
| POST BP Active | 2.03 | .886 |  |
| PRE MML Distressed | 3.97 | 1.014 |  |
| POST MML Distressed | 4.55 | .823 |  |
| PRE VML Distressed | 4.05 | .932 |  |
| POST VML Distressed | 4.54 | .662 |  |
| PRE BP Distressed | 4.29 | .851 |  |
| POST BP Distressed | 4.63 | .671 |  |
| PRE MML Upset | 3.99 | 1.049 |  |
| POST MML Upset | 4.35 | .912 |  |
| PRE VML Upset | 4.25 | .865 |  |
| POST VML Upset | 4.47 | .618 |  |
| PRE BP Upset | 4.32 | .887 |  |
| POST BP Upset | 4.60 | .633 |  |
| PRE MML Guilty | 4.50 | 1.072 |  |
| POST MML Guilty | 4.68 | .770 |  |
| PRE VML Guilty | 4.57 | .690 |  |
| POST VML Guilty | 4.63 | .697 |  |
| PRE BP Guilty | 4.80 | .536 |  |
| POST BP Guilty | 4.78 | .616 |  |
| PRE MML Afraid | 4.44 | 1.016 |  |
| POST MML Afraid | 4.71 | .800 |  |
| PRE VML Afraid | 4.54 | .691 |  |
| POST VML Afraid | 4.76 | .582 |  |
| PRE BP Afraid | 4.66 | .527 |  |
| POST BP Afraid | 4.88 | .350 |  |
| PRE MML Hostile | 4.35 | 1.153 |  |
| POST MML Hostile | 4.70 | .767 |  |
| PRE VML Hostile | 4.60 | .550 |  |
| POST VML Hostile | 4.55 | .744 |  |
| PRE BP Hostile | 4.79 | .426 |  |
| POST BP Hostile | 4.76 | .465 |  |
| PRE MML Irritable | 3.74 | 1.125 |  |
| POST MML Irritable | 4.57 | .872 |  |
| PRE VML Irritable | 4.03 | .941 |  |
| POST VML Irritable | 4.42 | .882 |  |
| PRE BP Irritable | 4.15 | .972 |  |
| POST BP Irritable | 4.65 | .671 |  |
| PRE MML Ashamed | 4.36 | 1.064 |  |
| POST MML Ashamed | 4.62 | .851 |  |
| PRE VML Ashamed | 4.51 | .751 |  |
| POST VML Ashamed | 4.77 | .582 |  |
| PRE BP Ashamed | 4.65 | .583 |  |
| POST BP Ashamed | 4.82 | .403 |  |
| PRE MML Nervous | 4.11 | 1.071 |  |
| POST MML Nervous | 4.56 | .786 |  |
| PRE VML Nervous | 3.88 | .980 |  |
| POST VML Nervous | 4.17 | .804 |  |
| PRE BP Nervous | 4.13 | .971 |  |
| POST BP Nervous | 4.14 | 1.014 |  |
| PRE MML Jittery | 3.90 | 1.185 |  |
| POST MML Jittery | 4.55 | .787 |  |
| PRE VML Jittery | 3.66 | 1.034 |  |
| POST VML Jittery | 4.25 | .772 |  |
| PRE BP Jittery | 4.21 | .868 |  |
| POST BP Jittery | 3.41 | 1.511 |  |
| PRE MML Scared | 4.38 | 1.039 |  |
| POST MML Scared | 4.72 | .740 |  |
| PRE VML Scared | 4.60 | .707 |  |
| POST VML Scared | 4.75 | .537 |  |
| PRE BP Scared | 4.69 | .494 |  |
| POST BP Scared | 4.91 | .229 |  |

*Appendix B*

| **Table 1**  *Principal effect of Musical Experience factor for item* | | | | | |
| --- | --- | --- | --- | --- | --- |
| ­­ Item | Source^a^ | F | df^b^ | p | η^2^ |
|  |  |  |  |  |  |
| Interested | Assumed sphericity | 12.457 | 2, 140 | .000* | .151 |
| Excited | Lower-bound | 10.395 | 1, 140 | .002* | .129 |
| Strong | Huynh-Feldt | 8.911 | 1.668,0.856 | .001* | .113 |
| Enthusiastic | Greenhouse-Geisser | 14.311 | 1.767, 123.716 | .000* | .170 |
| Proud | Huynh-Feldt | 5.041 | 1.771, 123.992 | .010* | .067 |
| Alert | Assumed sphericity | 60.656 | 2, 140 | .000* | .464 |
| Inspired | Assumed sphericity | 9.566 | 2, 140 | .000* | .120 |
| Determined | Huynh-Feldt | 4.110 | 1.606, 112.454 | .027* | .055 |
| Attentive | Huynh-Feldt | 3.378 | 1.876, 131.287 | .040* | .046 |
| Active | Assumed sphericity | 26.316 | 2, 140 | .000* | .273 |
| Distressed | Huynh-Feldt | 2.407 | 1.869, 130.825 | .098 | .033 |
| Upset | Huynh-Feldt | 4.133 | 1.569, 109.838 | .027* | .056 |
| Guilty | Huynh-Feldt | 2.584 | 1.779, 124.557 | .086 | .036 |
| Afraid | Huynh-Feldt | 2.745 | 1.842, 128.954 | .072 | .038 |
| Hostile | Huynh-Feldt | 4.160 | 1.681, 117.702 | .024* | .056 |
| Irritable | Assumed sphericity | 3.044 | 2, 140 | .051 | .042 |
| Ashamed | Huynh-Feldt | 3.245 | 1.637, 114.617 | .052 | .044 |
| Nervous | Huynh-Feldt | 3.600 | 1.716, 120.109 | .037* | .049 |
| Jittery | Huynh-Feldt | 3.749 | 1.891, 132.344 | .028* | .051 |
| Scared | Huynh-Feldt | 3.823 | 1.640, 114.825 | .032* | .052 |

*Note. ^a­^.* In *Source*, the selected correction (Greeenhouse-Geisser, Huynh-Feldt and Lower Bound) or no correction (Assumed sphericity) are based on the Mauchly’s test for sphericity.*^­^*

*^b.^* In *df*, the first term represents the degrees of freedom of the factor (Musical Experience) and the second one the degrees of freedom of the Error.

*. The effect is significant at the 0.05 level.

| **Table 2**  *Principal effect of Moment factor per item* | | | | | |
| --- | --- | --- | --- | --- | --- |
| ­­ Item | Source^a^ | F | df^b^ | p | η^2^ |
|  |  |  |  |  |  |
| Interested | Assumed sphericity | 14.340 | 1, 70 | .000* | .170 |
| Excited | Assumed sphericity | 43.552 | 1, 70 | .000* | .384 |
| Strong | Assumed sphericity | 10.161 | 1, 70 | .002* | .127 |
| Enthusiastic | Assumed sphericity | 26.440 | 1, 70 | .000* | .274 |
| Proud | Assumed sphericity | 16.807 | 1, 70 | .000* | .194 |
| Alert | Assumed sphericity | 11.391 | 1, 70 | .001* | .140 |
| Inspired | Assumed sphericity | 47.388 | 1, 70 | .000* | .404 |
| Determined | Assumed sphericity | 8.710 | 1, 70 | .004* | .111 |
| Attentive | Assumed sphericity | 38.730 | 1, 70 | .000* | .356 |
| Active | Assumed sphericity | 7.071 | 1, 70 | .010* | .092 |
| Distressed | Assumed sphericity | 42.268 | 1, 70 | .000* | .376 |
| Upset | Assumed sphericity | 20.035 | 1, 70 | .000* | .223 |
| Guilty | Assumed sphericity | 3.323 | 1, 70 | .073 | .045 |
| Afraid | Assumed sphericity | 29.078 | 1,70 | .000* | .293 |
| Hostile | Assumed sphericity | 2.438 | 1,70 | .123 | .034 |
| Irritable | Assumed sphericity | 62.735 | 1,70 | .000* | .473 |
| Ashamed | Assumed sphericity | 24.104 | 1,70 | .000* | .256 |
| Nervous | Assumed sphericity | 11.986 | 1,70 | .001* | .146 |
| Jittery | Assumed sphericity | 15.526 | 1,70 | .000* | .182 |
| Scared | Assumed sphericity | 31.637 | 1,70 | .000* | .311 |

*Note. ^a­^.* In *Source*, the selected correction (Greeenhouse-Geisser,and Lower Bound) or no correction (Assumed sphericity) are based on the Mauchly’s test for sphericity.*^­^*

*^b.^* In *df*, the first term represents the degrees of freedom of the factor (Musical Experience) and the second one the degrees of freedom of the Error.

*. The effect is significant at the 0.05 level.

| **Table 3**  *Principal effect of the interaction Musical Experience*Moment per item* | | | | | |
| --- | --- | --- | --- | --- | --- |
| Item | Source^a^ | F | df^b^ | p | η^2^ |
|  |  |  |  |  |  |
| Interested | Assumed sphericity | 13.830 | 2, 140 | .000* | .165 |
| Excited | Assumed sphericity | 13.741 | 2, 140 | .000* | .164 |
| Strong | Assumed sphericity | 1.058 | 2, 140 | .350 | .015 |
| Enthusiastic | Assumed sphericity | 11.490 | 2, 140 | .000* | .141 |
| Proud | Assumed sphericity | 6.034 | 2, 140 | .003* | .079 |
| Alert | Assumed sphericity | 43.893 | 2, 140 | .000* | .385 |
| Inspired | Assumed sphericity | 2.720 | 2, 140 | .069 | .037 |
| Determined | Assumed sphericity | 6.614 | 2, 140 | .002* | .086 |
| Attentive | Assumed sphericity | 0.859 | 2, 140 | .426 | .012 |
| Active | Assumed sphericity | 26.315 | 2, 140 | .000* | .273 |
| Distressed | Huynh-Feldt | 1.310 | 1.842, 128.919 | .272 | .018 |
| Upset | Assumed sphericity | 0.416 | 2, 140 | .660 | .006 |
| Guilty | Huynh-Feldt | 1.920 | 1.823, 127.605 | .155 | .027 |
| Afraid | Assumed sphericity | 0.132 | 2, 140 | .877 | .002 |
| Hostile | Huynh-Feldt | 7.143 | 1.890, 132.279 | .001* | .093 |
| Irritable | Assumed sphericity | 4.581 | 2, 140 | .012* | .061 |
| Ashamed | Assumed sphericity | 0.338 | 2, 140 | .714 | .005 |
| Nervous | Assumed sphericity | 4.184 | 2, 140 | .017* | .056 |
| Jittery | Assumed sphericity | 18.566 | 2, 140 | .000* | .210 |
| Scared | Huynh-Feldt | 1.518 | 1.780, 124.574 | .224 | .021 |

*Note. ^a­^.* In *Source*, the selected correction (Greeenhouse-Geisser and Lower Bound) or no correction (Assumed sphericity) are based on the Mauchly’s test for sphericity.

*^b.^* In *df*, the first term represents the degrees of freedom of the factor (Musical Experience) and the second one the degrees of freedom of the Error.

*. The effect is significant at the 0.05 level.

**Appendix C**

**Table 1**

*Pairwise comparisons of Musical Experience factor*

| Item | (I) Musical Experience | (J) Musical Experience | Mean Difference (I-J) | Std. Error | p | 95% Confidence Interval for Difference^b^ | |
| --- | --- | --- | --- | --- | --- | --- | --- |
|  |  |  |  |  |  | Lower Bound | Upper Bound |
| INTERESTED | 1 | 2 | .049 | .113 | 1.000 | -.228 | .325 |
|  |  | 3 | .466^*^ | .104 | .000 | .211 | .721 |
|  | 2 | 1 | -.049 | .113 | 1.000 | -.325 | .228 |
|  |  | 3 | .417^*^ | .090 | .000 | .197 | .638 |
|  | 3 | 1 | -.466^*^ | .104 | .000 | -.721 | -.211 |
|  |  | 2 | -.417^*^ | .090 | .000 | -.638 | -.197 |
| EXCITED | 1 | 2 | .245 | .125 | .162 | -.062 | .552 |
|  |  | 3 | .530^*^ | .135 | .001 | .200 | .861 |
|  | 2 | 1 | -.245 | .125 | .162 | -.552 | .062 |
|  |  | 3 | .285^*^ | .083 | .003 | .082 | .489 |
|  | 3 | 1 | -.530^*^ | .135 | .001 | -.861 | -.200 |
|  |  | 2 | -.285^*^ | .083 | .003 | -.489 | -.082 |
| STRONG | 1 | 2 | .260 | .117 | .090 | -.028 | .548 |
|  |  | 3 | .419^*^ | .104 | .000 | .165 | .674 |
|  | 2 | 1 | -.260 | .117 | .090 | -.548 | .028 |
|  |  | 3 | .159 | .075 | .112 | -.025 | .343 |
|  | 3 | 1 | -.419^*^ | .104 | .000 | -.674 | -.165 |
|  |  | 2 | -.159 | .075 | .112 | -.343 | .025 |
| ENTHUSIASTIC | 1 | 2 | .436^*^ | .128 | .003 | .121 | .751 |
|  |  | 3 | .621^*^ | .131 | .000 | .301 | .942 |
|  | 2 | 1 | -.436^*^ | .128 | .003 | -.751 | -.121 |
|  |  | 3 | .186 | .095 | .166 | -.048 | .419 |
|  | 3 | 1 | -.621^*^ | .131 | .000 | -.942 | -.301 |
|  |  | 2 | -.186 | .095 | .166 | -.419 | .048 |
| PROUD | 1 | 2 | -.015 | .112 | 1.000 | -.289 | .260 |
|  |  | 3 | .255^*^ | .092 | .021 | .030 | .479 |
|  | 2 | 1 | .015 | .112 | 1.000 | -.260 | .289 |
|  |  | 3 | .270^*^ | .080 | .004 | .073 | .466 |
|  | 3 | 1 | -.255^*^ | .092 | .021 | -.479 | -.030 |
|  |  | 2 | -.270^*^ | .080 | .004 | -.466 | -.073 |
| ALERT | 1 | 2 | 1.092^*^ | .109 | .000 | .825 | 1.360 |
|  |  | 3 | .956^*^ | .117 | .000 | .670 | 1.243 |
|  | 2 | 1 | -1.092^*^ | .109 | .000 | -1.360 | -.825 |
|  |  | 3 | -.136 | .098 | .503 | -.375 | .103 |
|  | 3 | 1 | -.956^*^ | .117 | .000 | -1.243 | -.670 |
|  |  | 2 | .136 | .098 | .503 | -.103 | .375 |
| INSPIRED | 1 | 2 | .290 | .130 | .088 | -.030 | .609 |
|  |  | 3 | .503^*^ | .108 | .000 | .237 | .769 |
|  | 2 | 1 | -.290 | .130 | .088 | -.609 | .030 |
|  |  | 3 | .213 | .106 | .145 | -.047 | .473 |
|  | 3 | 1 | -.503^*^ | .108 | .000 | -.769 | -.237 |
|  |  | 2 | -.213 | .106 | .145 | -.473 | .047 |
| DETERMINED | 1 | 2 | .197 | .100 | .157 | -.048 | .442 |
|  |  | 3 | .242 | .102 | .060 | -.007 | .491 |
|  | 2 | 1 | -.197 | .100 | .157 | -.442 | .048 |
|  |  | 3 | .045 | .062 | 1.000 | -.108 | .198 |
|  | 3 | 1 | -.242 | .102 | .060 | -.491 | .007 |
|  |  | 2 | -.045 | .062 | 1.000 | -.198 | .108 |
| ATTENTIVE | 1 | 2 | .168 | .102 | .320 | -.084 | .419 |
|  |  | 3 | .271 | .119 | .079 | -.022 | .563 |
|  | 2 | 1 | -.168 | .102 | .320 | -.419 | .084 |
|  |  | 3 | .103 | .092 | .796 | -.122 | .329 |
|  | 3 | 1 | -.271 | .119 | .079 | -.563 | .022 |
|  |  | 2 | -.103 | .092 | .796 | -.329 | .122 |
| ACTIVE | 1 | 2 | .531^*^ | .112 | .000 | .255 | .806 |
|  |  | 3 | .761^*^ | .116 | .000 | .478 | 1.045 |
|  | 2 | 1 | -.531^*^ | .112 | .000 | -.806 | -.255 |
|  |  | 3 | .230^*^ | .094 | .049 | .001 | .460 |
|  | 3 | 1 | -.761^*^ | .116 | .000 | -1.045 | -.478 |
|  |  | 2 | -.230^*^ | .094 | .049 | -.460 | -.001 |
| DISTRESSED | 1 | 2 | -.037 | .107 | 1.000 | -.300 | .226 |
|  |  | 3 | -.203 | .104 | .168 | -.458 | .053 |
|  | 2 | 1 | .037 | .107 | 1.000 | -.226 | .300 |
|  |  | 3 | -.166 | .082 | .139 | -.366 | .035 |
|  | 3 | 1 | .203 | .104 | .168 | -.053 | .458 |
|  |  | 2 | .166 | .082 | .139 | -.035 | .366 |
| UPSET | 1 | 2 | -.192 | .110 | .255 | -.463 | .078 |
|  |  | 3 | -.289 | .120 | .056 | -.583 | .005 |
|  | 2 | 1 | .192 | .110 | .255 | -.078 | .463 |
|  |  | 3 | -.096 | .070 | .518 | -.268 | .075 |
|  | 3 | 1 | .289 | .120 | .056 | -.005 | .583 |
|  |  | 2 | .096 | .070 | .518 | -.075 | .268 |
| GUILTY | 1 | 2 | -.015 | .104 | 1.000 | -.270 | .240 |
|  |  | 3 | -.203 | .113 | .228 | -.480 | .073 |
|  | 2 | 1 | .015 | .104 | 1.000 | -.240 | .270 |
|  |  | 3 | -.188 | .079 | .060 | -.382 | .005 |
|  | 3 | 1 | .203 | .113 | .228 | -.073 | .480 |
|  |  | 2 | .188 | .079 | .060 | -.005 | .382 |
| AFRAID | 1 | 2 | -.078 | .088 | 1.000 | -.295 | .139 |
|  |  | 3 | -.200 | .096 | .125 | -.436 | .037 |
|  | 2 | 1 | .078 | .088 | 1.000 | -.139 | .295 |
|  |  | 3 | -.121 | .071 | .276 | -.296 | .053 |
|  | 3 | 1 | .200 | .096 | .125 | -.037 | .436 |
|  |  | 2 | .121 | .071 | .276 | -.053 | .296 |
| HOSTILE | 1 | 2 | -.055 | .101 | 1.000 | -.302 | .192 |
|  |  | 3 | -.254 | .105 | .053 | -.510 | .003 |
|  | 2 | 1 | .055 | .101 | 1.000 | -.192 | .302 |
|  |  | 3 | -.199^*^ | .068 | .014 | -.365 | -.032 |
|  | 3 | 1 | .254 | .105 | .053 | -.003 | .510 |
|  |  | 2 | .199^*^ | .068 | .014 | .032 | .365 |
| IRRITABLE | 1 | 2 | -.076 | .108 | 1.000 | -.342 | .189 |
|  |  | 3 | -.243 | .107 | .080 | -.507 | .020 |
|  | 2 | 1 | .076 | .108 | 1.000 | -.189 | .342 |
|  |  | 3 | -.167 | .085 | .163 | -.376 | .042 |
|  | 3 | 1 | .243 | .107 | .080 | -.020 | .507 |
|  |  | 2 | .167 | .085 | .163 | -.042 | .376 |
| ASHAMED | 1 | 2 | -.150 | .111 | .539 | -.422 | .122 |
|  |  | 3 | -.246 | .107 | .072 | -.508 | .016 |
|  | 2 | 1 | .150 | .111 | .539 | -.122 | .422 |
|  |  | 3 | -.096 | .069 | .509 | -.266 | .074 |
|  | 3 | 1 | .246 | .107 | .072 | -.016 | .508 |
|  |  | 2 | .096 | .069 | .509 | -.074 | .266 |
| NERVOUS | 1 | 2 | .306^*^ | .121 | .042 | .008 | .604 |
|  |  | 3 | .202 | .133 | .405 | -.125 | .529 |
|  | 2 | 1 | -.306^*^ | .121 | .042 | -.604 | -.008 |
|  |  | 3 | -.105 | .089 | .725 | -.322 | .113 |
|  | 3 | 1 | -.202 | .133 | .405 | -.529 | .125 |
|  |  | 2 | .105 | .089 | .725 | -.113 | .322 |
| JITTERY | 1 | 2 | .271^*^ | .104 | .034 | .015 | .526 |
|  |  | 3 | -14.082^*^ | 1.264 | .000 | -17.18 | -10.98 |
|  | 2 | 1 | -.271^*^ | .104 | .034 | -.526 | -.015 |
|  |  | 3 | -14.352^*^ | 1.269 | .000 | -17.46 | -11.24 |
|  | 3 | 1 | 14.082^*^ | 1.264 | .000 | 10.98 | 17.18 |
|  |  | 2 | 14.352^*^ | 1.269 | .000 | 11.241 | 17.464 |
| SCARED | 1 | 2 | -.122 | .101 | .686 | -.369 | .125 |
|  |  | 3 | -.248^*^ | .100 | .046 | -.493 | -.003 |
|  | 2 | 1 | .122 | .101 | .686 | -.125 | .369 |
|  |  | 3 | -.126 | .064 | .158 | -.283 | .031 |
|  | 3 | 1 | .248^*^ | .100 | .046 | .003 | .493 |
|  |  | 2 | .126 | .064 | .158 | -.031 | .283 |
| *Note*. Based on marginal means | | | | | | | |
| *. Mean difference is significant at 0.05 level. | | | | | | | |
| b. Comparisons adjustment: Bonferroni. | | | | | | | |
